# Do Image-Assisted Mobile Applications Improve Dietary Habits, Knowledge, and Behaviours in Elite Athletes? A Pilot Study

**DOI:** 10.3390/sports5030060

**Published:** 2017-08-11

**Authors:** Anne Simpson, Luke Gemming, Dane Baker, Andrea Braakhuis

**Affiliations:** 1 Discipline of Nutrition and Dietetics, FM & HS, University of Auckland, Private Bag 92019, Auckland 1142, New Zealand; asim134@aucklanduni.ac.nz or anne.e.simpson@gmail.com; 2 School of Life and Environmental Services, University of Sydney, Sydney, New South Wales 2006, Australia; luke.gemming@sydney.com.au; 3 High Performance Sport, AUT Millenium, Auckland 0632, New Zealand; dane.baker@hpsnz.org.nz

**Keywords:** athlete, nutrition, m-health, sport, food-diary

## Abstract

To date, there has been a paucity of research on optimal ways to educate and promote dietary behavioural change within athletes. Optimising athlete nutrition is fundamental to reaching peak performance and maintaining athlete wellbeing. MealLogger^®^ is a smartphone application that incorporates the use of an image-based food record and social-media functionality to provide in-application personalised feedback to individuals or groups, peer-support, and a platform to deliver nutrition education material. This study measured the feasibility of MealLogger^®^ within New Zealand elite male field hockey players (*n* = 17) aged 18–20 to increase athlete knowledge and nutrition promoting behaviours. During a six-week intervention, participants were instructed to log images of their meals three days per week and they received individualised dietetic feedback on logged meals. Weekly nutrition-education fact-sheets and videos were delivered through the application. Nutrition knowledge increased moderately from baseline (%Pre 54.7 ± 14.3; %Post 61.1 ± 11.45, *p* = 0.01). Participants report a highly positive experience of application use (8/10) with 82.3% attempting to make positive changes in dietary behaviours based on in-app education. All participants preferred this method to traditional methods of dietary analysis. Using image-based applications such as MealLogger^®^ is an effective approach to monitor dietary intake and deliver education to optimise the nutritional behaviours of elite athletes.

## 1. Introduction

Elite athletes have unique nutritional demands to not only meet the energy needs of an extremely physical lifestyle but also to optimise nutrition delivery for high performance [1]. Poor nutrition has the ability to greatly impede athletic performance, and can result in deficiencies leading to metabolic upset, early fatigue, and increased risk of injury [2]. In order for sports dietitians to optimise athlete nutrition and meet athlete performance goals, it is generally accepted that a true and accurate reflection of dietary intake should be obtained. The current standard practice in dietary assessment of athletes is to request they record their dietary intake via pen and paper-based diary. The dietary records form the primary nutrition assessment are used to supplement other methods, such as a dietitian-led diet history. However, limitations using dietary records among athletes are evident and well known [3]. The burden for athletes to record dietary intake is exacerbated due to more frequent eating episodes or total food intake (increased energy requirements) in conjunction with busy training and work schedules, increasing opportunities and likelihood of underreporting [3]. Thus, the quality of the data obtained is typically far less than the desired goal to accurately analyse and optimise dietary intake for sport performance. Additionally, traditional dietary records are too burdensome for frequent recurrent assessments [4].

Participants clearly prefer electronic methods of dietary assessment compared to traditional approaches [4] and approximately one-third (32.4%) of sports dietitians (*n* = 180) already report using diet apps with their clients in a multi-nation cross-sectional survey [5]. Encouragingly, the majority of the dietitians rated diet apps as a very effective tool for improving client self-monitoring [5]. To date, a paucity of research has explored the use of image-based dietary monitoring apps or explored the potential of the in-app social media features to support dietetic care of athletes. Image-based dietary record apps are preferred by users, reduce recording burden, and aim to provide more accurate dietary intake data compared to traditional dietary records [6,7]. Image-based dietary records incorporating automated image-analysis techniques to estimate energy and nutrient intake are still undergoing development. Unfortunately, image-based dietary records require manual image analysis which is laborious and currently isolated to research applications only [8,9]. Thus, until automated methods are validated, image-based records can provide a simple means to monitor intake in real-time. 

In-app social media include features that provide a platform for sports dietitians to remotely monitor, educate and support behavior change for both individuals and groups of athletes. Viewing peer athlete or teammate meal-logs and making or reading posted comments may support team dynamics and cohesion, beyond nutrition knowledge. Education via apps may better support athletes residing in different regions where face-to-face contact is impractical. Studies using m-health apps with social-media capability increase the likelihood of clients meeting their nutrition goals through social support and enhancing self-efficacy, particularly when enrolled with like-minded individuals [10,11,12]. In-app social media features provide a simple method to deliver formal education material (information sheets, videos, or web-links) and the inbuilt data analytics report the statistics regarding views, duration of viewed content, and downloads. The inbuilt analytics provide objective feedback to dietitians regarding clients’ engagement and popularity of content. 

Limitations of traditional dietary assessment methods include under reporting, participant burden, short-comings in nutrient databases, and issues with standardising procedures. Considering the limitations of existing methods [5,13,14], alternative approaches, which do not attempt to quantitatively assess intake but rather monitor, maintain client contact, and encourage and educate athletes, are warranted. The aim of this research was to assess the feasibility of dietary education via mobile phone application use and the subsequent change in nutrition and sports nutrition knowledge. A secondary aim was to evaluate perceptions of behaviour change and willingness to further engage with sport dietitians on the basis of this pilot intervention.

## 2. Materials and Methods

This study consisted of 17 male field hockey players (mean ± SD, 19 ± 0.7 year). Participants involved in this study spent between 6 h and 23 h per week training for field hockey (mean ± SD, 12 ± 4.3 h∙wk^−1^). The majority of participants were residing in a living arrangement where people share rented accommodation 52%, with 35.3% living at home, 5.9% living at a university hostel, and 5.9% living in another form of residence. The majority of participants were currently at university 82.2% with 5.9% having obtained a degree and 5.9% working. All participants from this study were competitive at an International 94.1% or a National level 5.9%. The study was conducted in season leading up to a major competitive event. Participants were on the same team, but resided anywhere in the country for the duration of the intervention. Participants’ compliance, engagement, usability, and acceptance of MealLogger^®^ were evaluated through app-data analytics and a participant exit survey. The exit survey simply asked the participants to rate from 1 to 10 whether they felt their nutrition behaviors had improved and why.

Participants had access to a personal smart phone devices. Seventeen U21 New Zealand field hockey players were briefed in person on the details of the study away from influencing factors such as coaching staff and members of management. Anthropometric data (weight and skinfolds) were collected by the research team as subject characteristics. The field hockey players logged their meals three days a week over a six-week period and interacted with online video and fact sheet material. The days of recording changed each week to ensure an even distribution of data collection. The study was granted ethical approval by The University of Auckland Committee of Ethics (ref 016347).

MealLogger-Photo Food Journal^®^ (version 4.6, Wellness Foundry, Helsinki, Finland) was selected as the application of choice for this pilot study due to its unique platform for hosting a private group of individuals, having the ability to keep an image-based food diary, allowing researchers to post links to information sheets and videos as well as provide real time feedback. The MealLogger^®^ app used in the context of this study requires purchasing (group greater than 5). The app was introduced to players in the initial face to face meeting. All consenting participants received a step-by-step guide on how to download the MealLogger^®^ application, join the research group and log their meals. The research team downloaded MealLogger^®^ app from the Play Store prior to commencing the study to ensure iOS/android functionality and general usability.

Each week, participants received a link to a new fact-sheet shared through the MealLogger^®^ application. The fact sheets were produced based on education material gathered on current best practices [14,15,16,17,18,19,20,21,22,23,24] and in collaboration with sports dietetic expertise. The sheets were based on key sports nutrition advice regarding six selected topics targeting performance nutrition: hydration, body composition, supplements, dietary needs over a training week, recovery-nutrition, and game-day nutrition. Access to the education sheets was optional, allowing participants to conduct self-learning in topics which interested them each week. The fact sheets were one or two pages in length and represented a summary of the sport nutrition literature with simplified language with relevance to field hockey. The topics were selected by the dietitian with experience in sport nutrition for field hockey, with oversight from the research team. 

In addition to the fact sheets, each week participants received a link to a YouTube^TM^ (Mountain View, CA, USA) video presented to them through the MealLogger^®^ application. Videos were based around the weekly sports-nutrition initiatives, on the same topic detailed by the fact-sheets. Videos featured interview style questions between a researcher, an ex-New Zealand Hockey captain, and a leading sports dietitian. Inspiration for the videos was based on reinforcement of the sports dietetic expertise provided in the fact-sheets, allowing athletes to see how the nutrition principles could be practically applied in the real world. Similarly to the fact-sheets, viewing of these videos was optional. The videos ranged 1–3 min each. 

During the logging period, participants were provided with real-time sports-dietetic feedback. Feedback comments from the researcher were tailored to the posted meal received from the participant, giving specific nutrition advice, praising areas of positive behaviour and providing suggestions for future improvement. The nature of the feedback focused on the general serving size and diet quality, rather than gram intake of specific nutrients. Here is an example of feedback provided to a participant through the MealLogger^®^ app: “Awesome brekie! Full of fibre and complex carbs to give you slow burning energy across the day! Also nice to see you are having a good lean protein source (eggs) to aid muscle recovery & keeping on top of the hydration—Great pre-game meal choice.” Questions were prompted if any aspect of the meal consumed was unclear, such as preparation methods or quantity. Participants were encouraged to post as much as they desired including drinks and pre-post workout snacks. Participants could post-questions directly to team-mates within the group, or to the researcher publically or privately. The research team consisted of three dietitians and one student dietitian, with varying levels expertise in sport nutrition. Feedback was provided as quickly as practically possible, ranging from a few minutes to a few hours. The maximum timeframe by which feedback was provided was 8 h following the participant post. Example feedback posts were developed prior to study commencement to standardise the approach.

Participants completed one questionnaire, on two occasions, based on the previously validated nutritional knowledge questionnaire “Questionnaire of Nutritional Knowledge” [25]. The questionnaire construct, content, and re-test validity are described elsewhere [26,27], with a re-test correlation of 0.74–0.89 [27]. This questionnaire was designed to review (a) basic nutritional knowledge of the participants (11 questions); (b) behavioural effects of food availability and choice (8 questions); (c) sports nutrition knowledge and practices of the subjects (28 questions); and (d) demographic variables of the participants. The questionnaire was amended to include questions on current nutrition ideas within the media and questions specific to the education material provided. 

A pilot trial of the nutrition knowledge questionnaire was tested out on several elite hockey players of similar age and nutritional knowledge to amend the readability and appropriateness of before posting the questionnaire online for the participants to complete. The pilot study tested participants’ understanding and comprehension of the questionnaire. Five questions required slight rewording to clarify the request. Once the questionnaire was finalised, a link was sent to participants via email to enable participants to complete it in their own time, to the best of their ability, two weeks prior to the commencement of food logging. The questionnaire was converted to Qualtrics online platform and emailed to participants once consent was signed, prior to intervention (week -2) and immediately post intervention (week 7). Participants were emailed reminders to complete the survey.

### 2.1. Statistical Analysis

Questionnaire data presented as mean % score ± standard deviation. The questionnaire data was analysed using the paired *t*-test, following normality testing. Questionnaire results found to be significant were interpreted using Cohen’s effect sizes (0.2-small, 0.5 moderate) calculated using the inherent variability of the questionnaire data. The interpretation of the change in the questionnaire score is presented as qualitative statements with a reference to the magnitude of the change. The observed between-subject standard deviation was 8.2 in the questionnaire score, which we used to calculate the true standard deviation (SDTrue = $\sqrt{\left(\mathrm{SDObeserved}2\;-\;e2\right)}$ where e is the typical error). We multiplied the SDtrue by Cohen’s effects to estimate and apply qualitative statements that convey an interpretation of the magnitude of the change in the questionnaire data. The qualitative statements are defined as a change in the questionnaire data relating to ‘small’ > 1.2, ‘moderate’ > 2.9.

## 3. Results

Overall, the hockey players logged 577 meals over the course of the intervention. Compliance with logging meals started at 86% (week 1) and decreased to 59% (week 6); overall compliance was 66%. Participants appeared engaged with the app, with 444 comments or ‘likes’ generated on teammate’s posts. Nutrition professional support in the form of a comment or ‘like’ was provided for 90% of logged meals, median response time was 39 min (min = 2 min; max = 60 h 33 min).

Participants reported the education received resulted in positive dietary behaviour changes, 6/17 stating a significant improvement, 10/17 a moderate improvement, and 1/17 unsure. Many of the participants (13/17) attributed the positive changes due to viewing other team members’ meal-logs. 

Participants downloaded the body composition fact sheet and the hydration video more than other topics (see Table 1).

### 3.1. Nutrition Knowledge

Following the MealLogger^®^ nutrition intervention elite male hockey players observed significant improvements in nutrition knowledge, based on both sports nutrition and basic nutritional multi-choice questions, (Pre raw score 31.7, %Pre 54.7 ± 14.3; Post raw score 35.4, %Post 61.1 ± 11.45, *p* = 0.01). Given the small sample size and risk of a type-1 error, we interpreted the clinical significance and magnitude of the observed increase in test scores using Cohen’s effects, which showed a clear, moderate improvement in knowledge.

Sport nutrition specific topic knowledge improved. Broken down by topic, general nutrition knowledge (%Pre 58.8 ± 21.8; %Post 61.5 ± 24.6, *p* = 0.62); hydration (% Pre 61.4 ± 20.5, %Post 66.6 ± 16.08, *p* = 0.21); body composition (% Pre 29.4 ± 18.19; %Post 35.3 ± 24.2, *p* = 0.38); dietary supplements (%Pre 44.7 ± 26.01; %Post 52.9 ± 22.29, *p* = 0.31); recovery nutrition (%Pre 70.7 ± 14.6; %Post 52.9 ± 22.29, *p* = 0.83); event nutrition (%Pre 43.08 ± 14.62; %Post 51.6 ± 18.84, *p* = 0.17); training nutrition (%Pre 60.8 ± 29.44; %Post 76.5 ± 25.73, *p* = 0.05).

### 3.2. Application Usability

The participants’ experiences using MealLogger^®^ were largely positive with the mean participant rating of the experience of using the application being 8/10. The majority of participants (64.7%) rated the effort required to use the MealLogger^®^ application as a < 4 (on a 1–10 scale) indicating a fairly low associated burden. However, 35.3% of participants described application burden as > 6/10 with one reporting a 10/10 in regards to the effort required to log. The application usability was determined from the exit survey, which simply asked to what extent participants agreed (1–10) with statements regarding usability and behavior change which we have no variance data to report. 

To further investigate why some participants experienced a much higher burden of application use, issues with logging meals were investigated. Whilst 41.1% of participants “never” or “seldom” experienced any issues with the use of recording their diet via the MealLogger^®^ application, 35.5% sometimes experienced issues and 23.4% “often” or “always” experienced issues with this method. The described difficulties were based on the availability of data and Wi-Fi zones for participants to upload their meals. The second main issue described was accessibility of participants’ phones during meal times. Reported data indicates issues with the application itself were minimal with only one difficulty indicated, typing in the comments box to describe the participant’s meal. Other issues mentioned were the use of technology, and delaying meal consumption to capture images. 

Participants were asked how many weeks they deemed was acceptable to log meals as per our protocol. The majority of participants (64.7%) were comfortable using MealLogger^®^ for the entirety of the study with a mean time of six weeks. A further 41.2% also indicated they would be happy to continue to log their meals with MealLogger^®^ for a longer period of time than this study (>6 weeks to anytime). The minimum acceptable time reported in regards to participants feeling comfortable using MealLogger^®^ was three weeks (triple the time period of a three or four day diet history)

Despite some individuals facing issues logging their meals following the use of the application, every participant (100%) indicated that MealLogger^®^ was a preferable to record their dietary history as opposed to traditional paper based methods, or having no preference at all.

### 3.3. Nutrition Confidence and Behaviour Impact

Questionnaire results show a small increase in the mean self-rated nutrition knowledge (from 6.4 to 6.6) following MealLogger^®^ use. Participants also rated the mean increase in self-rated knowledge following the provision of personalised messages and comments 7.7/10. The mean self-rated impact on participant nutrition behaviour after receiving dietary information and education was 8/10. Overall, the majority of participants (82.3%) attempted to make some dietary changes based on the education material they received with only 17.6% of participants stating they made no dietary changes. All participants (100%) rated the process of logging their meals on having some impact on their behaviour with 64.7% of participants rated this process to have a relatively strong effect >6/10.

The majority of participants believed that viewing of their other teammates’ meals had an impact on their nutrition-related behavior, with 64.7% of participants rating a change in behaviour of 6/10 and above. Whilst 17.6% of participants rated the effect of viewing the meals of others as low or “did not change my behaviour”. 

Prior to the study 0% of participants stated they would prefer to receive individualised dietetic advice from a sports dietitian, improving to 82.4% post intervention.

## 4. Discussion

The primary objective of this study was to investigate if the mobile application MealLogger^®^ could be used as a platform to improve an athlete’s nutrition knowledge, monitor dietary intake, and positively influence dietary habits and behaviours using an image-based food record. Secondary objectives evaluated the athlete’s acceptance, engagement, and practicality using MealLogger^®^ in the six weeks leading to the elite hockey player’s national tournament. Overall feedback indicated that using MealLogger^®^ was a positive experience, enhanced the team environment, and positively impacted team dynamics. Improved sport-nutrition knowledge corroborated participant feedback (pre: 55% versus post: 61% mean correct answers, *p* = 0.01).

The use of MealLogger^®^ showed promise in promoting dietary behavioural change within athletes, which can be explained by the Social Cognitive Theory (SCT) [26]. The ability of an individual to self-reflect is a key component of the SCT model. People who exhibit high self-efficacy are confident in their ability to produce and change their own future, and are more likely to exhibit attempted and successful behavioural changes [28]. One method for increasing an individual’s feelings of self-efficacy is through reflection on self-behaviour [29]. Post-questionnaire results from this study revealed about two-thirds of participants believed the process of logging and observing their meals had a significant impact on their nutrition related behaviours. Additionally, there were no reports the application had a negative effect on dietary behaviour. The process of self-monitoring dietary intake itself has shown a number of positive benefits relating to health, such as by Hollis [30] which observed individuals aiming to lose weight exhibited twice as much weight loss, when completing a food diary than those who did not. Data analytics revealed the engagement with education material was highly variable depending on the week’s education theme.

The social media functionality of MealLogger^®^ provided a unique feature as it allowed both group members and dietitian to monitor dietary intake in near real time. Subsequent professional feedback by the dietitian can be personalised to the athletes requirements to optimise their dietary intake, or simply provide timely feedback e.g., a ‘like’ to encourage and support athletes efforts to consume a diet to achieve personal goals and/or optimise intake. Participants within this study rated receiving personalised comments and messages to have a high impact on dietary knowledge. The application enables athletes to reflect on their diet, based on immediate and professional feedback which is likely to further enhance self-efficacy. The dietetic feedback provides athletes with reassurance or assistance towards meeting their personal dietary goals.

Another aspect of the SCT employed within the design of this study, to enhance dietary behaviour change, was based on targeting expectations of the athletes [31]. The implementation of utilising a former New Zealand Black Sticks hockey captain as a role model, who demonstrated key nutrition skills through educational videos, was successful. Observing a role model who has achieved desirable international athletic results and exhibiting certain desirable nutrition behaviours, can positively influence the athlete’s forethought. Role modelling may too enhance the athletes own performance, if optimal nutrition behaviours are copied [31]. However, during this study, the video material views ranged from 4 to 11 depending on the education topic, therefore they were not accessed by every participant. More work is required on optimizing the use of role models in app related nutrition education.

Positive feedback from elite hockey players towards the acceptability of MealLogger^®^ as a tool for dietary monitoring and educating athletes reinforces the value of m-health tools. The social media component of this application observed significant unforeseen benefits for national teams located across the country, enabling participants to share meals together regardless of location, therefore enhancing the team environment. The social media component of this application also observed team support to increase the positive experience of logging a meal and provided meal ideas and inspiration for athletes to consume nutritious meals. This reinforces findings from studies using group therapy to enhance motivation and behavioural changes between like-minded individuals [32,33,34,35,36,37,38,39].

The use of technology with digital photography capabilities (such as mobile or personal digital assistant devices) has shown potential to increase the accuracy of dietary recording by capturing unreported foods, cooking methods, beverages, and condiments [40,41,42]. Future use of image-based dietary applications with social media platforms such as MealLogger^®^ allow for nutrition to come to the forefront of team initiatives with athletes having support and taking collective ownership for their dietary intake [10,11,12]. However, application usage is not limited to athletes involved in team sports and those involved in individual sports also have the potential to benefit from the group based aspect of applications such as MealLogger^®^ by linking in with other athletes and sharing experiences and meal ideas with athlete peers in order to reach their goals.

The major limitations to the current study were the lack of a control group and small sample size. Given the current investigation is a pilot study to determine feasibility, these studies require repetition to confirm the findings. A proportion of the nutrition knowledge questionnaire had been reworded to facilitate understanding from our participation group, however we cannot be sure how this has influenced the validity. Further consideration should surround how applications can enhance self-efficacy so athletes feel enabled to make positive dietary choices and behavioural changes. The provision of an application which allows for professionals to provide real-time individualised dietetic feedback based on a comprehensive image of an athlete’s daily intake such as MealLogger^®^ has been observed to be positively rated by elite athletes at promoting self-efficacy, and nutrition-related knowledge and behaviours. Positive dietary behaviours were also reinforced through group support, peer modelling, and the provision of resources. 

Future research should focus on how to maximise utilisation of these applications, optimal ways to provide education, and advice through these devices in a range of athlete populations. Quantification of the ability of these applications to capture a true image of athlete dietary intake may also be beneficial for all athletes. This pilot study however shows the promise of this application as a tool for the education and promotion of behavioural change in athletes, in a way which is highly preferable over traditional methods. 

## 5. Conclusions

The current study highlights the potential power of using mobile dietary applications to target education and behaviour change. To our knowledge, this pilot study was the first to use an image-assisted mobile phone application to educate and promote dietary behavioural changes within elite athletes. Nutrition knowledge was increased over the six-week intervention, and all participants described logging meals to have an impact on athlete behaviour. A key secondary finding was the improved attitude amongst participants to receiving advice from a sport dietitian. Prior to the study, 0% of participants stated they would prefer to receive individualised dietetic advice from a sports dietitian, improving to 82.4% post intervention. 

The mobile application MealLogger^®^ provided a unique platform to provide nutrition knowledge to athletes across New Zealand. Use of this application and online resources enhanced team-building by allowing participants to remain socially connected and essentially have meals together, regardless of location. The use of image-capture technology to assist dietary logging proved to be preferable for every athlete involved in this study over traditional methods of dietary assessment, and may aid in increasing compliance and decreasing the burden to participants. 

## Figures and Tables

**Table 1:** Participant engagement with education material.

Week	Topic	Views/Downloads of Fact Sheets	Views/Downloads of Education Videos
1	Hydration	6	11
2	Body composition	144	6
3	Supplements	52	7
4	Nutrition across a training week	2	7
5	Event nutrition	6	3
6	Nutrition for optimal recovery	4	4
